# Different intra- and inter­molecular hydrogen-bonding patterns in (3*S*,4a*S*,8a*S*)-2-[(2*R*,3*S*)-3-(2,5-*X*_2_-benzamido)-2-(2,5-*X*_2_-benzo­yloxy)-4-phenyl­butyl]-*N*-*tert*-butyldeca­hydro­iso­quinoline-3-carboxamides (*X* = H or Cl): compounds with moderate aspartyl protease inhibition activity

**DOI:** 10.1107/S2056989017007800

**Published:** 2017-05-31

**Authors:** Wilson Cunico, Maria de Lourdes G. Ferreira, James L. Wardell, William T. A. Harrison

**Affiliations:** a Departamento de Química Orgânica, Universidade Federal de Pelotas (UFPel), Campus Universitário, s/n, Caixa Postal 354, 96010-900 Pelotas, RS, Brazil; b Instituto de Tecnologia em Fármacos – Farmanguinhos, Fiocruz. R. Sizenando, Nabuco, 100, Manguinhos, 21041-250, Rio de Janeiro, RJ, Brazil; c Department of Chemistry, University of Aberdeen, Meston Walk, Aberdeen AB24 3UE, Scotland

**Keywords:** crystal structure, malaria, iso­quinoline­carboxamide, hydrogen bonding, aspartyl protease inhibition activity

## Abstract

The crystal structures of (3*S*,4a*S*,8a*S*)-2-[(2*R*,3*S*)-3-benzamido-2-benzo­yloxy-4-phenyl­but­yl]-*N*-*tert*-butyldeca­hydro­iso­quinoline-3-carboxamide, C_38_H_47_N_3_O_4_, (I), and (3*S*,4a*S*,8a*S*)-2-[(2*R*,3*S*)-3-(2,5-di­chloro­benzamido)-2-(2,5-di­chloro­benzo­yloxy)-4-phenyl­but­yl]-*N*-*tert*-butyldeca­hydro­iso­quinoline-3-carboxamide, C_38_H_43_Cl_4_N_3_O_4_, (II), are described. Despite their chemical similarity, they adopt different conformations in the solid state: (I) features a bifurcated intra­molecular N—H⋯(N,O) hydrogen bond from the *tert*-butylamide NH group to the piperidine N atom and the benzoate O atom, whereas (II) has an intra­molecular N—H⋯O link from the benzamide NH group to the *tert*-butyl­amide O atom. In the crystal of (I), mol­ecules are linked by *C*(4) amide N—H⋯O hydrogen bonds into chains propagating in the [010] direction, with both donor and acceptor parts of the benzamide group. In the extended structure of (II), *C*(11) N—H⋯O chains propagating in the [010] direction arise, with the donor being the *tert*-butylamide NH group and the acceptor being the O atom of the benzamide group.

## Abstract 2

The closely related title compounds show different intra- and inter­molecular hydrogen-bonding patterns.

## Chemical context   

Malaria remains one of the most devastating infectious diseases with over 200 million cases and more than 600 000 deaths each year – primarily children under the age of five in sub-Saharan Africa. There is an urgent need for effective drugs with new mechanisms of action, due to the high rate of mutation of the parasite, which leads to the development of resistance of current drugs.

One of the critical stages of the life cycle of the parasite during human infection is the degradation of haemoglobin, which provides nutrients for its growth and maturation (Coombs *et al.*, 2001 ▸). Plasmepsins are a family of aspartic proteases involved in the degradation of human haemoglobin by Plasmodium falciparum (Huizing *et al.*, 2015 ▸). As the parasite needs the resulting amino acid building blocks for its growth and development, plasmepsins are an important anti­malarial drug target. Secondary alcohols (Muthas *et al.*, 2005 ▸; Ersmark *et al.*, 2006 ▸) and tertiary alcohols (Motwani *et al.*, 2015 ▸) have been successfully used to develop potent inhibitors of these enzymes.

Cunico *et al.* (2008 ▸) reported the moderate *in vitro* anti­malarial activities of the products of reactions of the 2-amino­ethyl compound, **3** (see Scheme 1) with various sulfonyl chlorides and acyl chlorides. In the present article, we report the crystal structures of two compounds (see Scheme 2), C_38_H_47_N_3_O_4_, (I), and C_38_H_43_Cl_4_N_3_O_4_, (II), obtained in that study from reactions with acyl chlorides.
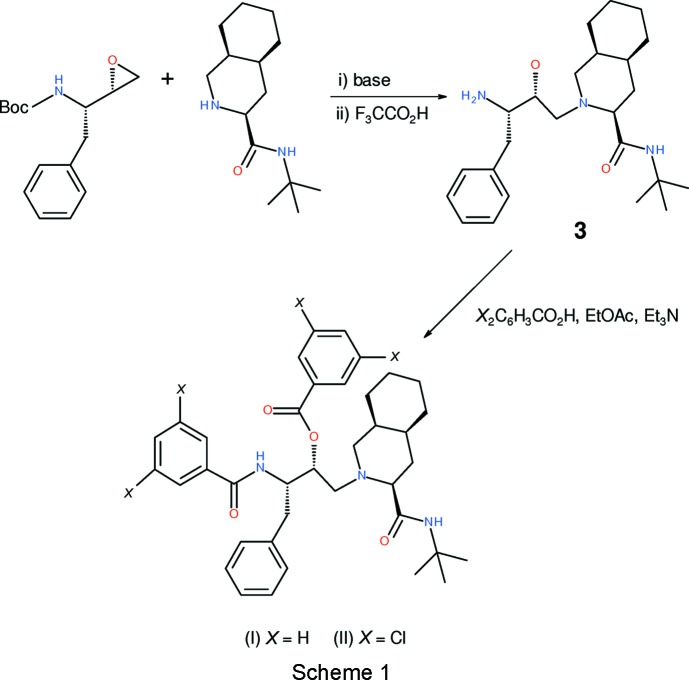


## Structural commentary   

**Figure 1:**
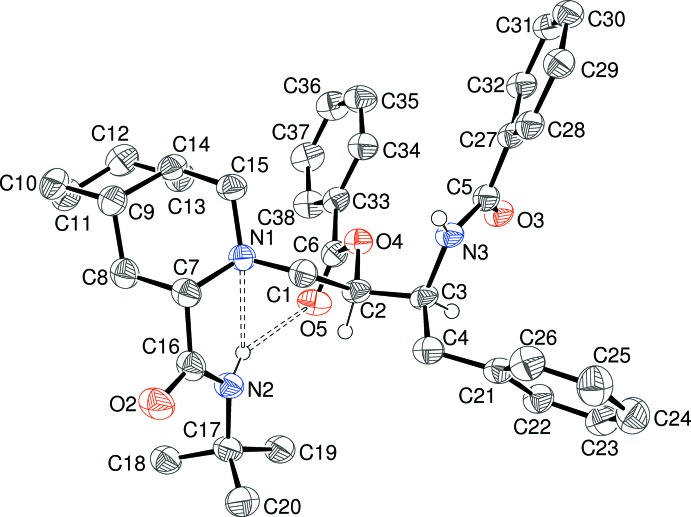
The asymmetric unit of (I), showing 50% probability displacement ellipsoids, with most H atoms omitted for clarity. The bifurcated intra­molecular hydrogen bond is shown as a double-dashed line.

Compound (I) crystallizes in the space group *P*2_1_ with a single mol­ecule in the asymmetric unit (Fig. 1 ▸). The absolute structure was not definitively established based on refinement of the Flack parameter (Parsons *et al.*, 2013 ▸) and the configurations of the stereogenic centres (C2 *R*, C3 *S*, C7 *S*, C9 *S*, C14 *S*) were set to match those in (II): they are those expected based on the known starting materials. Each atom in the C1—C2—C3—C4 ‘backbone’ of (I) bears a different substituent: C1 is attached to a piperidine+cyclo­hexane fused-ring system, which in turn bears a *tert*-butylamide group. C2 is attached to a benzoate group and C3 bears a benzamide group. Finally, C4 is attached to a simple phenyl ring, *i.e.* a benzyl group. Some key torsion angles are presented in Table 1 ▸. These show that with respect to the C2—C3 bond, the C1 + C4, C1 + N3 and N3 + O4 pairings are *gauche*, whereas the C4 + O4 atoms are mutually *anti*. In terms of the H atoms, H2 is *anti* to N3 (171°) and H3 is *anti* to C1 (176°); the *gauche* torsion angle between the H atoms is 54°. The N1—C1—C2—C3 torsion angle of 170.4 (3)° indicates an *anti* conformation and the N1/C7/C8/C9/C14/C5 and C9–C14 rings have a *cis*-fused junction (H9—C9—C14—H14 = −52°). The amide torsion angles C3—N3—C5—C27 and C17—N2—C16—C7 are −178.3 (3) and −164.7 (4)°, respectively, which reflect the expected near-planar conformations for these groups. The dihedral angles between the aromatic rings C21–C26 (*A*), C27–C32 (*B*) and C33–C38 (*C*) are *A*/*B* = 85.7 (2), *A*/*C* = 79.2 (2) and *B*/*C* = 17.3 (2)°. The conformation of (I) is supported by a bifurcated intra­molecular N—H⋯(N,O) hydrogen bond (Table 2 ▸) arising from the *tert*-butylamide group: the acceptor atoms are the N atom of the piperidine ring and the O atom of the C=O group of the benzoate group. The bifurcated bond is very asymmetric in terms of angles and the H⋯O link is long, but given that the assemblage is close to planar (bond-angle sum for the H atom = 353°), we regard it as being just significant.
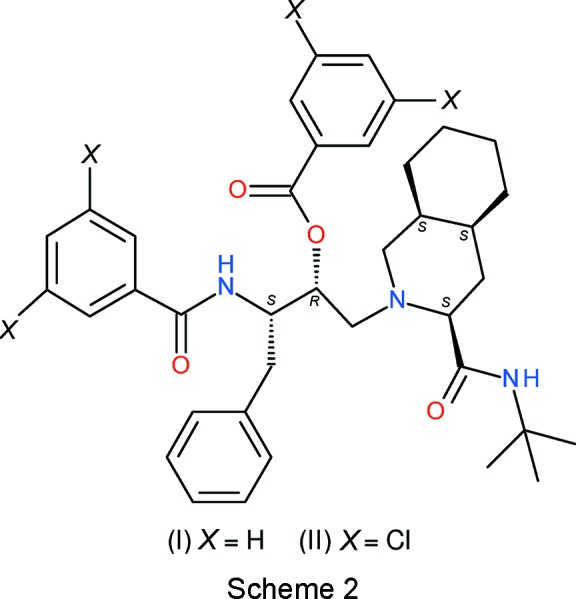


**Figure 2:**
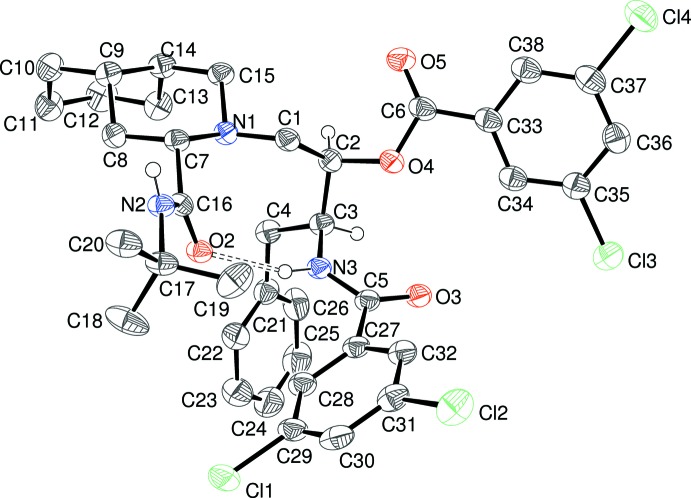
The asymmetric unit of (II), showing 50% probability displacement ellipsoids, with most H atoms omitted for clarity. The intra­molecular hydrogen bond is shown as a double-dashed line.

Compound (II) crystallizes in the space group *P*2_1_2_1_2_1_ with one mol­ecule in the asymmetric unit (Fig. 2 ▸). Here, the absolute structure is very well established (C2 *R*, C3 *S*, C7 *S*, C9 *S*, C14 *S*) and is consistent with the starting materials (Cunico *et al.*, 2008 ▸). The C1—C2—C3—C4 backbone bears the equivalent substituents to (I), with the difference that the benzyl and amide rings both bear a pair of Cl atoms at the *meta* positions. Selected torsion angles for (II) (Table 3 ▸) show similarities but also one major difference with respect to (I). In terms of the central C2—C3 bond in (II), the C1 + C4, C1 + N3 and N3 + O4 pairings are *gauche*, whereas the C4 + O4 atoms are mutually *anti*. With respect to the H atoms, H2 is *anti* to N3 (−175°) and H3 is *anti* to C1 (−166°); the torsion angle between the H atoms is 69°. Thus, the overall conformation of the atoms about the C2—C3 bond in (II) is essentially the same as in (I), although some of the torsion angles differ by as much as 20°. The N1—C1—C2—C3 *gauche* torsion angle of −69.1 (3)° in (II) is quite different to the value for (I) above, whereas the amide torsion angles C3—N3—C5—C27 [180.0 (3)°] and C17—N2—C16—C7 [–177.5 (3)°] in (II) are similar. The dihedral angles between the aromatic rings C21–C26 (*A*), C27–C32 (*B*) and C33–C38 (*C*) are *A*/*B* = 74.84 (17), *A*/*C* = 67.99 (17) and *B*/*C* = 68.91 (15)°: it may be seen that the first two of these values are similar to the equivalent data for (I), but the third value is very different, possibly reflecting a reorientation in (II) to minimize unfavourable steric inter­actions between the bichlorinated rings. Compound (II) features a completely different intra­molecular N—H⋯O hydrogen bond (Table 4 ▸) to (I): in (II), a much shorter (and presumably stronger) bond arises from the benzamide NH group to the *tert*-butylamide O atom, which no doubt correlates with the very different N1—C1—C2—C3 torsion angles for (I) and (II) already mentioned.

## Supra­molecular features   

**Figure 3:**
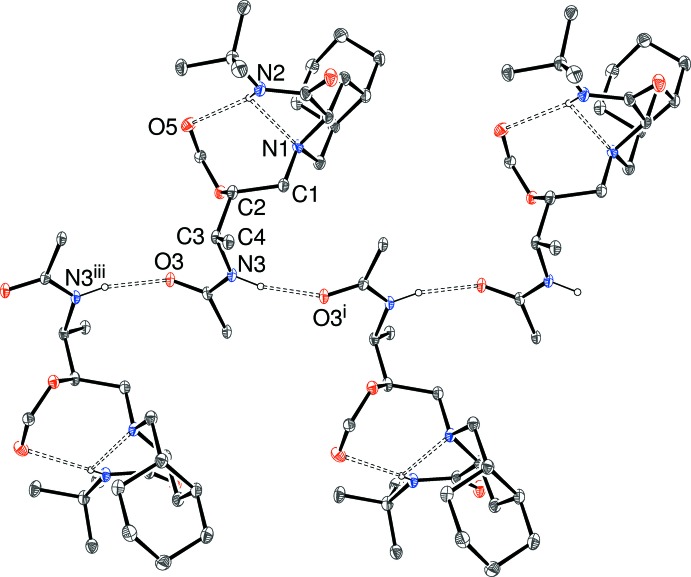
A fragment of a [010] hydrogen-bonded chain in (I), showing 20% probability displacement ellipsoids; the pendant rings and C-bound H atoms have been omitted for clarity. [Symmetry code as in Table 2 ▸; additionally (iii) −*x*, *y* − 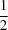, −*z*.]

In the crystal of (I), mol­ecules are linked by classical *C*(4) amide N—H⋯O hydrogen bonds into chains propagating in the [010] direction, with adjacent mol­ecules related by the 2_1_ screw axis. Both donor and acceptor are part of the benzamide group (Fig. 3 ▸). Two weak C—H⋯O inter­actions are also observed.

**Figure 4:**
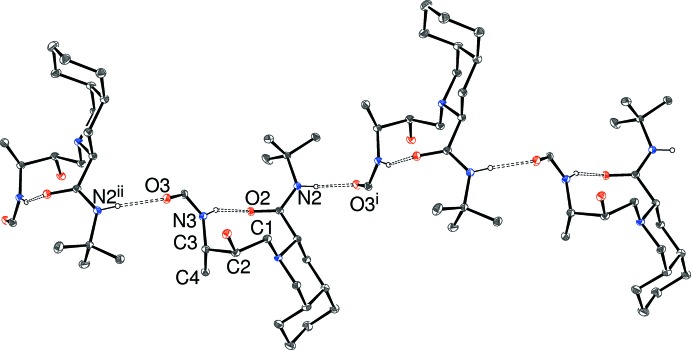
A fragment of a [010] hydrogen-bonded chain in (II), showing 20% probability displacement ellipsoids; the pendant rings and C-bound H atoms have been omitted for clarity. [Symmetry code as in Table 4 ▸; additionally (ii) −*x* + 1, *y* − 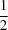, −*z* + 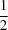.]

In the extended structure of (II), *C*(11) [010] N—H⋯O chains arise, with the donor being the *tert*-butylamide NH group and the acceptor being the O atom of the benzamide ring (Fig. 4 ▸). Adjacent mol­ecules are again related by a 2_1_ screw axis.

In short, for (I), the *tert*-butylamide NH moiety forms an intra­molecular hydrogen bond and the benzamide NH group forms an inter­molecular link, whereas for (II), the situation is reversed: the benzamide NH group forms the intra­molecular bond and the *tert*-butyl NH group forms the inter­molecular link.

## Database survey   

A survey of of the Cambridge Structural Database (Groom *et al.*, 2016 ▸: updated to April 2017) for the grouping of atoms about the C1—C2—C3—C4 fragment in (I) and (II) yielded 24 matches. The most similar are the isostructural halide salts YURSUB and YURTAI of the anti-HIV drug saquinavir mesylate (Fandaruff *et al.*, 2015 ▸), which also act as protease inhibitors. The other hits have little similarity to the title compounds.

## Synthesis and crystallisation   

As summarized in Scheme 1, compounds (I) and (II) were prepared as described previously (Cunico *et al.*, 2008 ▸) and recrystallized from methanol solution. (I): colourless needles, m.p. 475–476 K, ESI–HRMS (*M* + H): calculated for C_38_H_48_N_3_O_4_: 610.3645, found: 610.3638. (II): colourless slabs, m.p. 459–460 K, ESI–HRMS (*M* + H): calculated for C_38_H_44_^35^Cl_4_N_3_O_4_: 746.2086, found: 746.2078.

## Refinement   

Crystal data, data collection and structure refinement details are summarized in Table 5 ▸. The N-bound H atoms were located in difference maps and their positions were freely refined. The C-bound H atoms were placed geometrically (C—H = 0.95–1.00 Å) and refined as riding atoms. The constraint *U*_iso_(H) = 1.2*U*_eq_(C) or 1.5*U*_eq_(methyl C) was applied in all cases. The methyl groups were allowed to rotate, but not to tip, to best fit the electron density.

## Supplementary Material

Crystal structure: contains datablock(s) I, II, global. DOI: 10.1107/S2056989017007800/pk2602sup1.cif

Structure factors: contains datablock(s) I. DOI: 10.1107/S2056989017007800/pk2602Isup2.hkl

Structure factors: contains datablock(s) II. DOI: 10.1107/S2056989017007800/pk2602IIsup3.hkl

Supporting information file. DOI: 10.1107/S2056989017007800/pk2602Isup4.cml

Supporting information file. DOI: 10.1107/S2056989017007800/pk2602IIsup5.cml

CCDC references: 1552422, 1552421

Additional supporting information:  crystallographic information; 3D view; checkCIF report

## Figures and Tables

**Table 1:** Selected torsion angles (°) for (I)

N1—C1—C2—C3	170.4 (3)	C1—C2—C3—C4	59.4 (4)
C1—C2—C3—N3	−66.3 (4)	C4—C3—N3—C5	138.6 (4)
O4—C2—C3—C4	178.4 (3)	C3—C2—O4—C6	131.5 (3)

**Table 2:** Hydrogen-bond geometry (Å, °) for (I)

*D*—H⋯*A*	*D*—H	H⋯*A*	*D*⋯*A*	*D*—H⋯*A*
N2—H1*N*⋯O5	0.90 (5)	2.55 (5)	3.384 (5)	154 (4)
N2—H1*N*⋯N1	0.90 (5)	2.32 (5)	2.773 (4)	111 (4)
N3—H3*N*⋯O3^i^	0.93 (5)	2.04 (5)	2.929 (4)	161 (4)
C18—H18*B*⋯O2^ii^	0.98	2.39	3.310 (5)	157
C20—H20*A*⋯O2	0.98	2.35	2.963 (6)	120
C29—H29⋯O5^i^	0.95	2.58	3.467 (5)	157

Symmetry codes: (i) 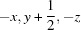; (ii) 
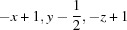
.

**Table 3:** Selected torsion angles (°) for (II)

N1—C1—C2—C3	−69.1 (3)	C1—C2—C3—C4	74.4 (3)
C1—C2—C3—N3	−49.5 (3)	C4—C3—N3—C5	136.6 (3)
O4—C2—C3—C4	−167.3 (2)	C3—C2—O4—C6	158.0 (2)

**Table 4:** Hydrogen-bond geometry (Å, °) for (II)

*D*—H⋯*A*	*D*—H	H⋯*A*	*D*⋯*A*	*D*—H⋯*A*
N2—H1*N*⋯O3^i^	0.84 (4)	2.13 (4)	2.931 (3)	160 (3)
N3—H2*N*⋯O2	0.88 (4)	1.99 (4)	2.834 (3)	159 (3)
C4—H4*A*⋯N1	0.99	2.55	3.149 (4)	119
C18—H18*A*⋯O2	0.98	2.36	2.975 (4)	120
C34—H34⋯O3	0.95	2.40	3.324 (4)	163

Symmetry code: (i) 
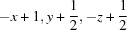
.

**Table 5:** Experimental details

	(I)	(II)
Crystal data		
Chemical formula	C_38_H_47_N_3_O_4_	C_38_H_43_Cl_4_N_3_O_4_
*M*_r_	609.78	747.55
Crystal system, space group	Monoclinic, *P*2_1_	Orthorhombic, *P*2_1_2_1_2_1_
Temperature (K)	100	100
*a*, *b*, *c* (Å)	11.4866 (3), 9.4448 (2), 16.8257 (5)	10.4539 (1), 15.1917 (1), 24.3677 (2)
α, β, γ (°)	90, 109.227 (3), 90	90, 90, 90
*V* (Å^3^)	1723.58 (8)	3869.90 (6)
*Z*	2	4
Radiation type	Cu *K*α	Cu *K*α
μ (mm^−1^)	0.60	3.12
Crystal size (mm)	0.52 × 0.15 × 0.05	0.25 × 0.20 × 0.04

Data collection		
Diffractometer	Rigaku Mercury CCD	Rigaku Mercury CCD
Absorption correction	Multi-scan (*SADABS*; Sheldrick, 2004 ▸)	Multi-scan (*SADABS*; Sheldrick, 2004 ▸)
*T*_min_, *T*_max_	0.654, 0.971	0.611, 0.886
No. of measured, independent and observed [*I* > 2σ(*I*)] reflections	24074, 5349, 4547	44109, 7278, 7140
*R*_int_	0.068	0.046
(sin θ/λ)_max_ (Å^−1^)	0.610	0.610

Refinement		
*R*[*F* ^2^ > 2σ(*F* ^2^)], *wR*(*F* ^2^), *S*	0.056, 0.151, 1.07	0.038, 0.100, 1.05
No. of reflections	5349	7278
No. of parameters	415	451
No. of restraints	1	0
H-atom treatment	H atoms treated by a mixture of independent and constrained refinement	H atoms treated by a mixture of independent and constrained refinement
Δρ_max_, Δρ_min_ (e Å^−3^)	0.35, −0.26	0.28, −0.32
Absolute structure	Flack *x* determined using 1316 quotients [(*I* ^+^) − (*I* ^−^)]/[(*I* ^+^) + (*I* ^−^)] (Parsons *et al.*, 2013 ▸)	Flack *x* determined using 3021 quotients [(*I* ^+^) − (*I* ^−^)]/[(*I* ^+^) + (*I* ^−^)] (Parsons *et al.*, 2013 ▸)
Absolute structure parameter	−0.4 (2)	−0.006 (7)

Computer programs: *CrysAlis PRO* (Rigaku, 2014 ▸), *SHELXS97* (Sheldrick, 2008 ▸), *SHELXL2014* (Sheldrick, 2015 ▸), *ORTEP-3 for Windows* (Farrugia, 2012 ▸) and *publCIF* (Westrip, 2010 ▸).

## supplementary crystallographic information

### (I) (3S,4aS,8aS)-2-[(2R,3S)-3-Benzamido-2-benzoyloxy-4-phenylbutyl]-N-tert-butyldecahydroisoquinoline-3-carboxamide Crystal data

**Table:**

C_38_H_47_N_3_O_4_	*F*(000) = 656
*M**_r_* = 609.78	*D*_x_ = 1.175 Mg m^−^^3^
Monoclinic, *P*2_1_	Cu *K*α radiation, λ = 1.54184 Å
*a* = 11.4866 (3) Å	Cell parameters from 8813 reflections
*b* = 9.4448 (2) Å	θ = 5.4–69.6°
*c* = 16.8257 (5) Å	µ = 0.60 mm^−^^1^
β = 109.227 (3)°	*T* = 100 K
*V* = 1723.58 (8) Å^3^	Needle, colourless
*Z* = 2	0.52 × 0.15 × 0.05 mm

### (I) (3S,4aS,8aS)-2-[(2R,3S)-3-Benzamido-2-benzoyloxy-4-phenylbutyl]-N-tert-butyldecahydroisoquinoline-3-carboxamide Data collection

**Table:**

Rigaku Mercury CCD diffractometer	4547 reflections with *I* > 2σ(*I*)
ω scans	*R*_int_ = 0.068
Absorption correction: multi-scan (SADABS; Sheldrick, 2004)	θ_max_ = 70.1°, θ_min_ = 2.8°
*T*_min_ = 0.654, *T*_max_ = 0.971	*h* = −14→13
24074 measured reflections	*k* = −11→9
5349 independent reflections	*l* = −20→19

### (I) (3S,4aS,8aS)-2-[(2R,3S)-3-Benzamido-2-benzoyloxy-4-phenylbutyl]-N-tert-butyldecahydroisoquinoline-3-carboxamide Refinement

**Table:**

Refinement on *F*^2^	Hydrogen site location: mixed
Least-squares matrix: full	H atoms treated by a mixture of independent and constrained refinement
*R*[*F*^2^ > 2σ(*F*^2^)] = 0.056	*w* = 1/[σ^2^(*F*_o_^2^) + (0.0894*P*)^2^ + 0.2672*P*] where *P* = (*F*_o_^2^ + 2*F*_c_^2^)/3
*wR*(*F*^2^) = 0.151	(Δ/σ)_max_ < 0.001
*S* = 1.07	Δρ_max_ = 0.35 e Å^−^^3^
5349 reflections	Δρ_min_ = −0.26 e Å^−^^3^
415 parameters	Absolute structure: Flack *x* determined using 1316 quotients [(I+)-(I-)]/[(I+)+(I-)] (Parsons *et al.*, 2013)
1 restraint	Absolute structure parameter: −0.4 (2)
Primary atom site location: structure-invariant direct methods	

### (I) (3S,4aS,8aS)-2-[(2R,3S)-3-Benzamido-2-benzoyloxy-4-phenylbutyl]-N-tert-butyldecahydroisoquinoline-3-carboxamide Special details

**Table:**

Geometry. All esds (except the esd in the dihedral angle between two l.s. planes) are estimated using the full covariance matrix. The cell esds are taken into account individually in the estimation of esds in distances, angles and torsion angles; correlations between esds in cell parameters are only used when they are defined by crystal symmetry. An approximate (isotropic) treatment of cell esds is used for estimating esds involving l.s. planes.

### (I) (3S,4aS,8aS)-2-[(2R,3S)-3-Benzamido-2-benzoyloxy-4-phenylbutyl]-N-tert-butyldecahydroisoquinoline-3-carboxamide Fractional atomic coordinates and isotropic or equivalent isotropic displacement parameters (Å^2^)

**Table:**

	*x*	*y*	*z*	*U*_iso_*/*U*_eq_	
C1	0.1094 (4)	0.3195 (4)	0.1956 (2)	0.0347 (8)	
H1A	0.1950	0.3530	0.2069	0.042*	
H1B	0.0566	0.3672	0.1438	0.042*	
C2	0.1046 (3)	0.1585 (4)	0.1806 (2)	0.0322 (8)	
H2	0.1668	0.1113	0.2295	0.039*	
C3	0.1262 (3)	0.1138 (4)	0.0997 (2)	0.0310 (8)	
H3	0.1265	0.0079	0.0988	0.037*	
C4	0.2513 (3)	0.1625 (4)	0.0978 (2)	0.0353 (8)	
H4A	0.2491	0.2665	0.0900	0.042*	
H4B	0.3140	0.1414	0.1530	0.042*	
C5	−0.0678 (3)	0.0764 (4)	−0.0162 (2)	0.0322 (8)	
C6	−0.0309 (4)	0.0410 (4)	0.2415 (2)	0.0344 (9)	
C7	0.1555 (3)	0.4553 (4)	0.3252 (2)	0.0362 (8)	
H7	0.1668	0.5406	0.2933	0.043*	
C8	0.1086 (4)	0.5037 (5)	0.3955 (3)	0.0408 (9)	
H8A	0.1674	0.5733	0.4311	0.049*	
H8B	0.1060	0.4213	0.4312	0.049*	
C9	−0.0194 (4)	0.5709 (5)	0.3631 (3)	0.0408 (9)	
H9	−0.0135	0.6587	0.3314	0.049*	
C10	−0.0665 (4)	0.6127 (5)	0.4343 (3)	0.0469 (10)	
H10A	−0.1408	0.6726	0.4114	0.056*	
H10B	−0.0027	0.6698	0.4759	0.056*	
C11	−0.0984 (4)	0.4852 (5)	0.4784 (3)	0.0510 (12)	
H11A	−0.0224	0.4307	0.5069	0.061*	
H11B	−0.1330	0.5179	0.5218	0.061*	
C12	−0.1913 (4)	0.3899 (5)	0.4161 (3)	0.0497 (11)	
H12A	−0.2702	0.4415	0.3917	0.060*	
H12B	−0.2072	0.3054	0.4457	0.060*	
C13	−0.1435 (4)	0.3435 (5)	0.3454 (3)	0.0430 (10)	
H13A	−0.0704	0.2821	0.3691	0.052*	
H13B	−0.2079	0.2871	0.3038	0.052*	
C14	−0.1088 (4)	0.4686 (5)	0.3014 (2)	0.0381 (9)	
H14	−0.1863	0.5225	0.2732	0.046*	
C15	−0.0557 (3)	0.4256 (4)	0.2330 (3)	0.0379 (9)	
H15A	−0.1128	0.3580	0.1944	0.046*	
H15B	−0.0501	0.5105	0.1999	0.046*	
C16	0.2805 (4)	0.3896 (5)	0.3683 (3)	0.0389 (9)	
C17	0.3889 (4)	0.1660 (5)	0.4317 (3)	0.0395 (9)	
C18	0.4024 (4)	0.1975 (5)	0.5232 (3)	0.0430 (10)	
H18A	0.4144	0.2995	0.5336	0.064*	
H18B	0.4737	0.1462	0.5604	0.064*	
H18C	0.3277	0.1673	0.5345	0.064*	
C19	0.3574 (4)	0.0085 (5)	0.4131 (3)	0.0484 (11)	
H19A	0.2764	−0.0113	0.4182	0.073*	
H19B	0.4200	−0.0499	0.4533	0.073*	
H19C	0.3556	−0.0135	0.3557	0.073*	
C20	0.5072 (4)	0.1970 (6)	0.4129 (3)	0.0516 (12)	
H20A	0.5284	0.2973	0.4235	0.077*	
H20B	0.4952	0.1751	0.3538	0.077*	
H20C	0.5742	0.1386	0.4492	0.077*	
C21	0.2909 (3)	0.0951 (4)	0.0296 (2)	0.0345 (8)	
C22	0.3112 (4)	−0.0514 (5)	0.0303 (3)	0.0381 (9)	
H22	0.2960	−0.1089	0.0721	0.046*	
C23	0.3533 (4)	−0.1124 (5)	−0.0299 (3)	0.0449 (10)	
H23	0.3680	−0.2115	−0.0282	0.054*	
C24	0.3743 (4)	−0.0325 (6)	−0.0919 (3)	0.0508 (11)	
H24	0.4034	−0.0756	−0.1328	0.061*	
C25	0.3523 (4)	0.1131 (6)	−0.0941 (3)	0.0523 (11)	
H25	0.3657	0.1698	−0.1369	0.063*	
C26	0.3109 (4)	0.1745 (5)	−0.0337 (3)	0.0444 (10)	
H26	0.2959	0.2736	−0.0358	0.053*	
C27	−0.1602 (3)	0.1360 (4)	−0.0933 (2)	0.0315 (8)	
C28	−0.1291 (4)	0.2311 (4)	−0.1467 (2)	0.0335 (8)	
H28	−0.0470	0.2653	−0.1323	0.040*	
C29	−0.2175 (4)	0.2756 (4)	−0.2206 (3)	0.0370 (9)	
H29	−0.1955	0.3395	−0.2568	0.044*	
C30	−0.3380 (4)	0.2273 (4)	−0.2417 (3)	0.0386 (9)	
H30	−0.3984	0.2582	−0.2923	0.046*	
C31	−0.3700 (4)	0.1339 (4)	−0.1889 (3)	0.0382 (9)	
H31	−0.4525	0.1011	−0.2033	0.046*	
C32	−0.2825 (3)	0.0883 (4)	−0.1156 (3)	0.0353 (8)	
H32	−0.3052	0.0240	−0.0798	0.042*	
C33	−0.1626 (3)	0.0098 (4)	0.2289 (2)	0.0346 (8)	
C34	−0.2557 (4)	0.0418 (5)	0.1540 (3)	0.0400 (9)	
H34	−0.2354	0.0806	0.1081	0.048*	
C35	−0.3772 (4)	0.0176 (5)	0.1463 (3)	0.0481 (11)	
H35	−0.4406	0.0405	0.0952	0.058*	
C36	−0.4071 (4)	−0.0400 (5)	0.2126 (3)	0.0493 (11)	
H36	−0.4910	−0.0566	0.2068	0.059*	
C37	−0.3155 (4)	−0.0735 (5)	0.2872 (3)	0.0506 (11)	
H37	−0.3363	−0.1127	0.3328	0.061*	
C38	−0.1931 (4)	−0.0496 (5)	0.2950 (3)	0.0449 (10)	
H38	−0.1298	−0.0738	0.3458	0.054*	
N1	0.0677 (3)	0.3598 (3)	0.26662 (19)	0.0332 (7)	
N2	0.2845 (3)	0.2467 (4)	0.3755 (2)	0.0369 (8)	
H1N	0.208 (4)	0.209 (6)	0.358 (3)	0.044*	
N3	0.0279 (3)	0.1602 (3)	0.02500 (19)	0.0301 (7)	
H3N	0.027 (4)	0.252 (6)	0.005 (3)	0.036*	
O2	0.3711 (3)	0.4674 (3)	0.3992 (2)	0.0506 (8)	
O3	−0.0799 (2)	−0.0457 (3)	0.00716 (17)	0.0344 (6)	
O4	−0.0177 (2)	0.1111 (3)	0.17533 (16)	0.0329 (6)	
O5	0.0540 (2)	0.0080 (4)	0.30361 (18)	0.0449 (7)	

### (I) (3S,4aS,8aS)-2-[(2R,3S)-3-Benzamido-2-benzoyloxy-4-phenylbutyl]-N-tert-butyldecahydroisoquinoline-3-carboxamide Atomic displacement parameters (Å^2^)

**Table:**

	*U*^11^	*U*^22^	*U*^33^	*U*^12^	*U*^13^	*U*^23^
C1	0.036 (2)	0.0253 (19)	0.040 (2)	−0.0010 (16)	0.0092 (16)	0.0009 (15)
C2	0.0261 (17)	0.0251 (19)	0.043 (2)	0.0008 (15)	0.0086 (15)	0.0016 (16)
C3	0.0292 (17)	0.0212 (18)	0.0410 (19)	0.0052 (15)	0.0094 (14)	0.0021 (15)
C4	0.0318 (18)	0.027 (2)	0.045 (2)	−0.0015 (16)	0.0095 (15)	0.0010 (17)
C5	0.0328 (19)	0.023 (2)	0.039 (2)	−0.0003 (15)	0.0090 (15)	−0.0032 (15)
C6	0.037 (2)	0.030 (2)	0.038 (2)	0.0008 (16)	0.0138 (17)	0.0014 (16)
C7	0.037 (2)	0.0251 (19)	0.042 (2)	−0.0047 (16)	0.0062 (16)	0.0014 (17)
C8	0.042 (2)	0.031 (2)	0.045 (2)	−0.0043 (18)	0.0092 (17)	−0.0029 (17)
C9	0.045 (2)	0.027 (2)	0.047 (2)	0.0004 (18)	0.0100 (18)	0.0000 (17)
C10	0.050 (2)	0.032 (2)	0.057 (3)	0.006 (2)	0.015 (2)	−0.005 (2)
C11	0.057 (3)	0.049 (3)	0.048 (2)	0.008 (2)	0.020 (2)	−0.002 (2)
C12	0.052 (3)	0.043 (3)	0.059 (3)	0.002 (2)	0.024 (2)	0.005 (2)
C13	0.043 (2)	0.033 (2)	0.053 (2)	−0.0001 (18)	0.0147 (18)	−0.0012 (19)
C14	0.035 (2)	0.033 (2)	0.044 (2)	0.0068 (17)	0.0097 (16)	0.0013 (17)
C15	0.033 (2)	0.032 (2)	0.045 (2)	0.0014 (16)	0.0073 (16)	0.0013 (16)
C16	0.039 (2)	0.030 (2)	0.043 (2)	−0.0064 (17)	0.0076 (17)	−0.0028 (16)
C17	0.0319 (19)	0.033 (2)	0.046 (2)	0.0001 (17)	0.0033 (16)	−0.0008 (18)
C18	0.041 (2)	0.035 (2)	0.047 (2)	0.0005 (18)	0.0055 (17)	0.0029 (18)
C19	0.044 (2)	0.030 (2)	0.059 (3)	0.0076 (19)	0.001 (2)	−0.004 (2)
C20	0.041 (2)	0.051 (3)	0.060 (3)	0.003 (2)	0.013 (2)	−0.004 (2)
C21	0.0264 (17)	0.032 (2)	0.043 (2)	−0.0004 (16)	0.0082 (14)	0.0002 (17)
C22	0.036 (2)	0.031 (2)	0.047 (2)	−0.0002 (17)	0.0132 (16)	−0.0019 (18)
C23	0.036 (2)	0.036 (2)	0.060 (3)	−0.0019 (18)	0.0131 (19)	−0.007 (2)
C24	0.048 (2)	0.050 (3)	0.057 (3)	−0.008 (2)	0.020 (2)	−0.011 (2)
C25	0.059 (3)	0.055 (3)	0.050 (2)	−0.006 (2)	0.026 (2)	0.001 (2)
C26	0.047 (2)	0.035 (2)	0.052 (2)	−0.0017 (19)	0.0174 (18)	0.003 (2)
C27	0.0311 (18)	0.0192 (18)	0.042 (2)	0.0015 (15)	0.0095 (15)	−0.0018 (15)
C28	0.0353 (19)	0.0226 (18)	0.041 (2)	0.0007 (16)	0.0099 (16)	−0.0031 (16)
C29	0.043 (2)	0.023 (2)	0.042 (2)	0.0030 (16)	0.0095 (17)	0.0019 (16)
C30	0.038 (2)	0.028 (2)	0.043 (2)	0.0039 (17)	0.0031 (17)	−0.0025 (17)
C31	0.0310 (18)	0.031 (2)	0.048 (2)	0.0012 (16)	0.0063 (16)	−0.0010 (17)
C32	0.0333 (19)	0.0219 (19)	0.048 (2)	−0.0015 (16)	0.0099 (16)	−0.0005 (16)
C33	0.0347 (19)	0.0245 (18)	0.046 (2)	−0.0012 (16)	0.0156 (16)	0.0015 (16)
C34	0.038 (2)	0.034 (2)	0.047 (2)	−0.0014 (17)	0.0115 (17)	0.0035 (18)
C35	0.036 (2)	0.045 (3)	0.059 (3)	−0.004 (2)	0.0104 (19)	0.003 (2)
C36	0.040 (2)	0.043 (3)	0.065 (3)	−0.008 (2)	0.018 (2)	0.004 (2)
C37	0.047 (2)	0.047 (3)	0.062 (3)	−0.002 (2)	0.024 (2)	0.010 (2)
C38	0.042 (2)	0.042 (3)	0.051 (2)	−0.001 (2)	0.0156 (18)	0.008 (2)
N1	0.0307 (16)	0.0262 (17)	0.0396 (17)	−0.0022 (13)	0.0075 (13)	−0.0028 (13)
N2	0.0325 (16)	0.0274 (18)	0.0445 (18)	0.0014 (14)	0.0040 (14)	0.0008 (14)
N3	0.0289 (15)	0.0188 (16)	0.0398 (16)	0.0015 (12)	0.0075 (12)	0.0012 (13)
O2	0.0407 (16)	0.0357 (17)	0.0652 (19)	−0.0084 (14)	0.0038 (14)	−0.0029 (15)
O3	0.0356 (13)	0.0185 (13)	0.0470 (15)	0.0024 (11)	0.0111 (11)	0.0009 (11)
O4	0.0303 (12)	0.0283 (14)	0.0391 (13)	−0.0033 (11)	0.0100 (10)	0.0022 (11)
O5	0.0361 (15)	0.0494 (18)	0.0457 (16)	0.0009 (14)	0.0087 (12)	0.0121 (14)

### (I) (3S,4aS,8aS)-2-[(2R,3S)-3-Benzamido-2-benzoyloxy-4-phenylbutyl]-N-tert-butyldecahydroisoquinoline-3-carboxamide Geometric parameters (Å, º)

**Table:**

C1—N1	1.476 (5)	C17—N2	1.472 (5)
C1—C2	1.539 (5)	C17—C20	1.522 (6)
C1—H1A	0.9900	C17—C18	1.526 (6)
C1—H1B	0.9900	C17—C19	1.538 (6)
C2—O4	1.449 (4)	C18—H18A	0.9800
C2—C3	1.521 (5)	C18—H18B	0.9800
C2—H2	1.0000	C18—H18C	0.9800
C3—N3	1.453 (4)	C19—H19A	0.9800
C3—C4	1.519 (5)	C19—H19B	0.9800
C3—H3	1.0000	C19—H19C	0.9800
C4—C21	1.507 (6)	C20—H20A	0.9800
C4—H4A	0.9900	C20—H20B	0.9800
C4—H4B	0.9900	C20—H20C	0.9800
C5—O3	1.241 (5)	C21—C26	1.383 (6)
C5—N3	1.346 (5)	C21—C22	1.403 (6)
C5—C27	1.490 (5)	C22—C23	1.383 (6)
C6—O5	1.213 (5)	C22—H22	0.9500
C6—O4	1.346 (5)	C23—C24	1.372 (7)
C6—C33	1.486 (5)	C23—H23	0.9500
C7—N1	1.465 (5)	C24—C25	1.397 (8)
C7—C16	1.512 (6)	C24—H24	0.9500
C7—C8	1.522 (6)	C25—C26	1.383 (7)
C7—H7	1.0000	C25—H25	0.9500
C8—C9	1.527 (6)	C26—H26	0.9500
C8—H8A	0.9900	C27—C28	1.397 (5)
C8—H8B	0.9900	C27—C32	1.403 (5)
C9—C10	1.520 (6)	C28—C29	1.386 (5)
C9—C14	1.537 (6)	C28—H28	0.9500
C9—H9	1.0000	C29—C30	1.388 (6)
C10—C11	1.522 (7)	C29—H29	0.9500
C10—H10A	0.9900	C30—C31	1.384 (6)
C10—H10B	0.9900	C30—H30	0.9500
C11—C12	1.520 (7)	C31—C32	1.380 (6)
C11—H11A	0.9900	C31—H31	0.9500
C11—H11B	0.9900	C32—H32	0.9500
C12—C13	1.529 (6)	C33—C38	1.390 (6)
C12—H12A	0.9900	C33—C34	1.391 (5)
C12—H12B	0.9900	C34—C35	1.378 (6)
C13—C14	1.516 (6)	C34—H34	0.9500
C13—H13A	0.9900	C35—C36	1.382 (6)
C13—H13B	0.9900	C35—H35	0.9500
C14—C15	1.524 (6)	C36—C37	1.384 (7)
C14—H14	1.0000	C36—H36	0.9500
C15—N1	1.479 (5)	C37—C38	1.387 (6)
C15—H15A	0.9900	C37—H37	0.9500
C15—H15B	0.9900	C38—H38	0.9500
C16—O2	1.240 (5)	N2—H1N	0.90 (5)
C16—N2	1.354 (5)	N3—H3N	0.93 (5)
			
N1—C1—C2	112.5 (3)	N2—C17—C18	109.8 (3)
N1—C1—H1A	109.1	C20—C17—C18	111.8 (3)
C2—C1—H1A	109.1	N2—C17—C19	106.4 (3)
N1—C1—H1B	109.1	C20—C17—C19	108.1 (4)
C2—C1—H1B	109.1	C18—C17—C19	109.5 (4)
H1A—C1—H1B	107.8	C17—C18—H18A	109.5
O4—C2—C3	107.4 (3)	C17—C18—H18B	109.5
O4—C2—C1	107.3 (3)	H18A—C18—H18B	109.5
C3—C2—C1	114.4 (3)	C17—C18—H18C	109.5
O4—C2—H2	109.2	H18A—C18—H18C	109.5
C3—C2—H2	109.2	H18B—C18—H18C	109.5
C1—C2—H2	109.2	C17—C19—H19A	109.5
N3—C3—C4	111.1 (3)	C17—C19—H19B	109.5
N3—C3—C2	112.4 (3)	H19A—C19—H19B	109.5
C4—C3—C2	111.7 (3)	C17—C19—H19C	109.5
N3—C3—H3	107.1	H19A—C19—H19C	109.5
C4—C3—H3	107.1	H19B—C19—H19C	109.5
C2—C3—H3	107.1	C17—C20—H20A	109.5
C21—C4—C3	114.4 (3)	C17—C20—H20B	109.5
C21—C4—H4A	108.7	H20A—C20—H20B	109.5
C3—C4—H4A	108.7	C17—C20—H20C	109.5
C21—C4—H4B	108.7	H20A—C20—H20C	109.5
C3—C4—H4B	108.7	H20B—C20—H20C	109.5
H4A—C4—H4B	107.6	C26—C21—C22	118.1 (4)
O3—C5—N3	122.8 (3)	C26—C21—C4	121.7 (4)
O3—C5—C27	120.3 (3)	C22—C21—C4	120.1 (4)
N3—C5—C27	116.9 (3)	C23—C22—C21	120.2 (4)
O5—C6—O4	124.2 (4)	C23—C22—H22	119.9
O5—C6—C33	124.3 (4)	C21—C22—H22	119.9
O4—C6—C33	111.5 (3)	C24—C23—C22	121.3 (4)
N1—C7—C16	113.8 (3)	C24—C23—H23	119.4
N1—C7—C8	111.5 (3)	C22—C23—H23	119.4
C16—C7—C8	105.9 (3)	C23—C24—C25	119.0 (4)
N1—C7—H7	108.5	C23—C24—H24	120.5
C16—C7—H7	108.5	C25—C24—H24	120.5
C8—C7—H7	108.5	C26—C25—C24	119.8 (5)
C7—C8—C9	113.1 (3)	C26—C25—H25	120.1
C7—C8—H8A	109.0	C24—C25—H25	120.1
C9—C8—H8A	109.0	C25—C26—C21	121.5 (4)
C7—C8—H8B	109.0	C25—C26—H26	119.2
C9—C8—H8B	109.0	C21—C26—H26	119.2
H8A—C8—H8B	107.8	C28—C27—C32	118.7 (3)
C10—C9—C8	112.2 (3)	C28—C27—C5	122.9 (3)
C10—C9—C14	111.3 (4)	C32—C27—C5	118.3 (3)
C8—C9—C14	109.3 (3)	C29—C28—C27	120.3 (4)
C10—C9—H9	108.0	C29—C28—H28	119.9
C8—C9—H9	108.0	C27—C28—H28	119.9
C14—C9—H9	108.0	C28—C29—C30	120.3 (4)
C9—C10—C11	112.6 (4)	C28—C29—H29	119.9
C9—C10—H10A	109.1	C30—C29—H29	119.9
C11—C10—H10A	109.1	C31—C30—C29	119.8 (4)
C9—C10—H10B	109.1	C31—C30—H30	120.1
C11—C10—H10B	109.1	C29—C30—H30	120.1
H10A—C10—H10B	107.8	C32—C31—C30	120.3 (4)
C12—C11—C10	111.0 (4)	C32—C31—H31	119.9
C12—C11—H11A	109.4	C30—C31—H31	119.9
C10—C11—H11A	109.4	C31—C32—C27	120.6 (4)
C12—C11—H11B	109.4	C31—C32—H32	119.7
C10—C11—H11B	109.4	C27—C32—H32	119.7
H11A—C11—H11B	108.0	C38—C33—C34	119.5 (4)
C11—C12—C13	110.8 (4)	C38—C33—C6	118.6 (3)
C11—C12—H12A	109.5	C34—C33—C6	121.9 (4)
C13—C12—H12A	109.5	C35—C34—C33	120.1 (4)
C11—C12—H12B	109.5	C35—C34—H34	119.9
C13—C12—H12B	109.5	C33—C34—H34	119.9
H12A—C12—H12B	108.1	C34—C35—C36	120.2 (4)
C14—C13—C12	112.1 (4)	C34—C35—H35	119.9
C14—C13—H13A	109.2	C36—C35—H35	119.9
C12—C13—H13A	109.2	C35—C36—C37	120.4 (4)
C14—C13—H13B	109.2	C35—C36—H36	119.8
C12—C13—H13B	109.2	C37—C36—H36	119.8
H13A—C13—H13B	107.9	C36—C37—C38	119.5 (4)
C13—C14—C15	113.3 (4)	C36—C37—H37	120.2
C13—C14—C9	112.6 (3)	C38—C37—H37	120.2
C15—C14—C9	109.4 (3)	C37—C38—C33	120.3 (4)
C13—C14—H14	107.0	C37—C38—H38	119.8
C15—C14—H14	107.0	C33—C38—H38	119.8
C9—C14—H14	107.0	C7—N1—C1	111.6 (3)
N1—C15—C14	113.2 (3)	C7—N1—C15	111.0 (3)
N1—C15—H15A	108.9	C1—N1—C15	108.9 (3)
C14—C15—H15A	108.9	C16—N2—C17	124.8 (3)
N1—C15—H15B	108.9	C16—N2—H1N	111 (3)
C14—C15—H15B	108.9	C17—N2—H1N	120 (3)
H15A—C15—H15B	107.7	C5—N3—C3	122.5 (3)
O2—C16—N2	123.6 (4)	C5—N3—H3N	117 (3)
O2—C16—C7	119.5 (4)	C3—N3—H3N	121 (3)
N2—C16—C7	116.8 (3)	C6—O4—C2	118.2 (3)
N2—C17—C20	111.1 (4)		
			
N1—C1—C2—O4	51.4 (4)	N3—C5—C27—C32	−151.0 (4)
N1—C1—C2—C3	170.4 (3)	C32—C27—C28—C29	−0.7 (6)
O4—C2—C3—N3	52.7 (4)	C5—C27—C28—C29	175.6 (4)
C1—C2—C3—N3	−66.3 (4)	C27—C28—C29—C30	0.7 (6)
O4—C2—C3—C4	178.4 (3)	C28—C29—C30—C31	−0.2 (6)
C1—C2—C3—C4	59.4 (4)	C29—C30—C31—C32	−0.2 (6)
N3—C3—C4—C21	−66.6 (4)	C30—C31—C32—C27	0.1 (6)
C2—C3—C4—C21	167.0 (3)	C28—C27—C32—C31	0.3 (6)
N1—C7—C8—C9	−54.5 (5)	C5—C27—C32—C31	−176.2 (3)
C16—C7—C8—C9	−178.9 (3)	O5—C6—C33—C38	6.1 (6)
C7—C8—C9—C10	177.6 (4)	O4—C6—C33—C38	−173.6 (4)
C7—C8—C9—C14	53.6 (5)	O5—C6—C33—C34	−175.7 (4)
C8—C9—C10—C11	−70.2 (5)	O4—C6—C33—C34	4.6 (6)
C14—C9—C10—C11	52.6 (5)	C38—C33—C34—C35	1.3 (7)
C9—C10—C11—C12	−55.8 (5)	C6—C33—C34—C35	−176.8 (4)
C10—C11—C12—C13	56.2 (5)	C33—C34—C35—C36	−0.6 (7)
C11—C12—C13—C14	−55.1 (5)	C34—C35—C36—C37	0.1 (8)
C12—C13—C14—C15	177.6 (3)	C35—C36—C37—C38	−0.3 (8)
C12—C13—C14—C9	52.7 (5)	C36—C37—C38—C33	1.0 (7)
C10—C9—C14—C13	−51.0 (5)	C34—C33—C38—C37	−1.5 (7)
C8—C9—C14—C13	73.5 (4)	C6—C33—C38—C37	176.7 (4)
C10—C9—C14—C15	−178.0 (3)	C16—C7—N1—C1	−63.7 (4)
C8—C9—C14—C15	−53.6 (4)	C8—C7—N1—C1	176.5 (3)
C13—C14—C15—N1	−69.4 (4)	C16—C7—N1—C15	174.5 (3)
C9—C14—C15—N1	57.3 (4)	C8—C7—N1—C15	54.8 (4)
N1—C7—C16—O2	159.3 (4)	C2—C1—N1—C7	130.4 (3)
C8—C7—C16—O2	−77.9 (5)	C2—C1—N1—C15	−106.6 (3)
N1—C7—C16—N2	−25.0 (5)	C14—C15—N1—C7	−57.9 (4)
C8—C7—C16—N2	97.9 (4)	C14—C15—N1—C1	178.8 (3)
C3—C4—C21—C26	117.9 (4)	O2—C16—N2—C17	10.8 (7)
C3—C4—C21—C22	−63.6 (5)	C7—C16—N2—C17	−164.7 (4)
C26—C21—C22—C23	1.7 (6)	C20—C17—N2—C16	−57.7 (6)
C4—C21—C22—C23	−176.8 (3)	C18—C17—N2—C16	66.5 (5)
C21—C22—C23—C24	−1.0 (6)	C19—C17—N2—C16	−175.1 (4)
C22—C23—C24—C25	−0.1 (7)	O3—C5—N3—C3	1.0 (6)
C23—C24—C25—C26	0.5 (7)	C27—C5—N3—C3	−178.3 (3)
C24—C25—C26—C21	0.2 (7)	C4—C3—N3—C5	138.6 (4)
C22—C21—C26—C25	−1.3 (6)	C2—C3—N3—C5	−95.4 (4)
C4—C21—C26—C25	177.2 (4)	O5—C6—O4—C2	−4.2 (6)
O3—C5—C27—C28	−146.7 (4)	C33—C6—O4—C2	175.5 (3)
N3—C5—C27—C28	32.6 (5)	C3—C2—O4—C6	131.5 (3)
O3—C5—C27—C32	29.7 (5)	C1—C2—O4—C6	−105.0 (4)

### (I) (3S,4aS,8aS)-2-[(2R,3S)-3-Benzamido-2-benzoyloxy-4-phenylbutyl]-N-tert-butyldecahydroisoquinoline-3-carboxamide Hydrogen-bond geometry (Å, º)

**Table:**

*D*—H···*A*	*D*—H	H···*A*	*D*···*A*	*D*—H···*A*
N2—H1*N*···O5	0.90 (5)	2.55 (5)	3.384 (5)	154 (4)
N2—H1*N*···N1	0.90 (5)	2.32 (5)	2.773 (4)	111 (4)
N3—H3*N*···O3^i^	0.93 (5)	2.04 (5)	2.929 (4)	161 (4)
C18—H18*B*···O2^ii^	0.98	2.39	3.310 (5)	157
C20—H20*A*···O2	0.98	2.35	2.963 (6)	120
C29—H29···O5^i^	0.95	2.58	3.467 (5)	157

Symmetry codes: (i) −*x*, *y*+1/2, −*z*; (ii) −*x*+1, *y*−1/2, −*z*+1.

### (II) (3S,4aS,8aS)-2-[(2R,3S)-3-(2,5-Dichlorobenzamido)-2-(2,5-dichlorobenzoyloxy)-4-phenylbutyl]-N-tert-butyldecahydroisoquinoline-3-carboxamide Crystal data

**Table:**

C_38_H_43_Cl_4_N_3_O_4_	*D*_x_ = 1.283 Mg m^−^^3^
*M**_r_* = 747.55	Cu *K*α radiation, λ = 1.54184 Å
Orthorhombic, *P*2_1_2_1_2_1_	Cell parameters from 40379 reflections
*a* = 10.4539 (1) Å	θ = 3.4–70.0°
*b* = 15.1917 (1) Å	µ = 3.12 mm^−^^1^
*c* = 24.3677 (2) Å	*T* = 100 K
*V* = 3869.90 (6) Å^3^	Slab, colourless
*Z* = 4	0.25 × 0.20 × 0.04 mm
*F*(000) = 1568	

### (II) (3S,4aS,8aS)-2-[(2R,3S)-3-(2,5-Dichlorobenzamido)-2-(2,5-dichlorobenzoyloxy)-4-phenylbutyl]-N-tert-butyldecahydroisoquinoline-3-carboxamide Data collection

**Table:**

Rigaku Mercury CCD diffractometer	7140 reflections with *I* > 2σ(*I*)
ω scans	*R*_int_ = 0.046
Absorption correction: multi-scan (SADABS; Sheldrick, 2004)	θ_max_ = 70.1°, θ_min_ = 3.4°
*T*_min_ = 0.611, *T*_max_ = 0.886	*h* = −12→12
44109 measured reflections	*k* = −15→18
7278 independent reflections	*l* = −29→29

### (II) (3S,4aS,8aS)-2-[(2R,3S)-3-(2,5-Dichlorobenzamido)-2-(2,5-dichlorobenzoyloxy)-4-phenylbutyl]-N-tert-butyldecahydroisoquinoline-3-carboxamide Refinement

**Table:**

Refinement on *F*^2^	Hydrogen site location: mixed
Least-squares matrix: full	H atoms treated by a mixture of independent and constrained refinement
*R*[*F*^2^ > 2σ(*F*^2^)] = 0.038	*w* = 1/[σ^2^(*F*_o_^2^) + (0.0552*P*)^2^ + 1.8039*P*] where *P* = (*F*_o_^2^ + 2*F*_c_^2^)/3
*wR*(*F*^2^) = 0.100	(Δ/σ)_max_ < 0.001
*S* = 1.05	Δρ_max_ = 0.28 e Å^−^^3^
7278 reflections	Δρ_min_ = −0.32 e Å^−^^3^
451 parameters	Absolute structure: Flack *x* determined using 3021 quotients [(I+)-(I-)]/[(I+)+(I-)] (Parsons *et al.*, 2013)
0 restraints	Absolute structure parameter: −0.006 (7)
Primary atom site location: structure-invariant direct methods	

### (II) (3S,4aS,8aS)-2-[(2R,3S)-3-(2,5-Dichlorobenzamido)-2-(2,5-dichlorobenzoyloxy)-4-phenylbutyl]-N-tert-butyldecahydroisoquinoline-3-carboxamide Special details

**Table:**

Geometry. All esds (except the esd in the dihedral angle between two l.s. planes) are estimated using the full covariance matrix. The cell esds are taken into account individually in the estimation of esds in distances, angles and torsion angles; correlations between esds in cell parameters are only used when they are defined by crystal symmetry. An approximate (isotropic) treatment of cell esds is used for estimating esds involving l.s. planes.

### (II) (3S,4aS,8aS)-2-[(2R,3S)-3-(2,5-Dichlorobenzamido)-2-(2,5-dichlorobenzoyloxy)-4-phenylbutyl]-N-tert-butyldecahydroisoquinoline-3-carboxamide Fractional atomic coordinates and isotropic or equivalent isotropic displacement parameters (Å^2^)

**Table:**

	*x*	*y*	*z*	*U*_iso_*/*U*_eq_	
C1	0.3339 (3)	0.3116 (2)	0.28983 (12)	0.0291 (6)	
H1A	0.4228	0.3055	0.2764	0.035*	
H1B	0.2948	0.3629	0.2712	0.035*	
C2	0.2587 (3)	0.2289 (2)	0.27568 (12)	0.0300 (6)	
H2	0.1702	0.2335	0.2910	0.036*	
C3	0.3188 (3)	0.1407 (2)	0.29311 (12)	0.0291 (6)	
H3	0.2717	0.0929	0.2734	0.035*	
C4	0.3109 (3)	0.1206 (2)	0.35483 (13)	0.0330 (7)	
H4A	0.3595	0.1657	0.3755	0.040*	
H4B	0.2205	0.1235	0.3669	0.040*	
C5	0.4901 (3)	0.09795 (19)	0.22901 (12)	0.0283 (6)	
C6	0.1687 (3)	0.2720 (2)	0.18833 (14)	0.0339 (7)	
C7	0.4476 (3)	0.37726 (19)	0.36694 (12)	0.0269 (6)	
H7	0.4450	0.4368	0.3495	0.032*	
C8	0.4452 (3)	0.3883 (2)	0.42941 (12)	0.0304 (6)	
H8A	0.4500	0.3297	0.4471	0.036*	
H8B	0.5209	0.4228	0.4411	0.036*	
C9	0.3234 (3)	0.4354 (2)	0.44809 (13)	0.0327 (7)	
H9	0.3238	0.4952	0.4309	0.039*	
C10	0.3151 (4)	0.4484 (2)	0.51013 (14)	0.0399 (7)	
H10A	0.3968	0.4735	0.5235	0.048*	
H10B	0.2464	0.4913	0.5183	0.048*	
C11	0.2880 (4)	0.3630 (3)	0.54077 (14)	0.0455 (8)	
H11A	0.2758	0.3760	0.5802	0.055*	
H11B	0.3625	0.3232	0.5372	0.055*	
C12	0.1691 (4)	0.3171 (3)	0.51853 (16)	0.0503 (9)	
H12A	0.0930	0.3542	0.5257	0.060*	
H12B	0.1571	0.2603	0.5378	0.060*	
C13	0.1815 (3)	0.3005 (2)	0.45668 (14)	0.0407 (8)	
H13A	0.2534	0.2596	0.4498	0.049*	
H13B	0.1021	0.2727	0.4428	0.049*	
C14	0.2048 (3)	0.3865 (2)	0.42625 (13)	0.0347 (7)	
H14	0.1289	0.4252	0.4327	0.042*	
C15	0.2175 (3)	0.3736 (2)	0.36460 (13)	0.0318 (6)	
H15A	0.1428	0.3399	0.3511	0.038*	
H15B	0.2164	0.4319	0.3464	0.038*	
C16	0.5715 (3)	0.33129 (19)	0.35033 (12)	0.0255 (6)	
C17	0.7959 (3)	0.3617 (2)	0.31935 (14)	0.0311 (6)	
C18	0.8574 (3)	0.2958 (3)	0.3576 (2)	0.0518 (10)	
H18A	0.8061	0.2418	0.3584	0.078*	
H18B	0.8622	0.3208	0.3947	0.078*	
H18C	0.9439	0.2821	0.3446	0.078*	
C19	0.7847 (4)	0.3250 (3)	0.26108 (16)	0.0478 (9)	
H19A	0.7327	0.2714	0.2616	0.072*	
H19B	0.8702	0.3112	0.2470	0.072*	
H19C	0.7441	0.3689	0.2373	0.072*	
C20	0.8752 (3)	0.4461 (2)	0.31835 (15)	0.0369 (7)	
H20A	0.8331	0.4897	0.2949	0.055*	
H20B	0.9606	0.4333	0.3038	0.055*	
H20C	0.8829	0.4694	0.3557	0.055*	
C21	0.3644 (4)	0.0305 (2)	0.36758 (13)	0.0378 (7)	
C22	0.4947 (4)	0.0212 (3)	0.38043 (15)	0.0451 (8)	
H22	0.5464	0.0723	0.3843	0.054*	
C23	0.5494 (5)	−0.0614 (3)	0.38766 (17)	0.0558 (10)	
H23	0.6376	−0.0664	0.3965	0.067*	
C24	0.4763 (6)	−0.1352 (3)	0.38198 (17)	0.0620 (12)	
H24	0.5144	−0.1916	0.3858	0.074*	
C25	0.3478 (5)	−0.1284 (3)	0.37081 (18)	0.0601 (12)	
H25	0.2969	−0.1800	0.3679	0.072*	
C26	0.2912 (4)	−0.0445 (2)	0.36356 (16)	0.0503 (9)	
H26	0.2024	−0.0400	0.3559	0.060*	
C27	0.6317 (3)	0.10128 (19)	0.21805 (13)	0.0296 (6)	
C28	0.7207 (3)	0.0856 (2)	0.25959 (14)	0.0335 (7)	
H28	0.6934	0.0722	0.2958	0.040*	
C29	0.8505 (3)	0.0899 (2)	0.24673 (15)	0.0363 (7)	
C30	0.8922 (3)	0.1106 (2)	0.19463 (16)	0.0385 (7)	
H30	0.9810	0.1148	0.1867	0.046*	
C31	0.8013 (3)	0.1254 (2)	0.15382 (15)	0.0365 (7)	
C32	0.6711 (3)	0.12013 (19)	0.16489 (13)	0.0318 (6)	
H32	0.6100	0.1293	0.1366	0.038*	
C33	0.1663 (3)	0.2464 (2)	0.12927 (13)	0.0331 (7)	
C34	0.2152 (3)	0.1651 (2)	0.11265 (14)	0.0341 (7)	
H34	0.2583	0.1278	0.1379	0.041*	
C35	0.1993 (3)	0.1402 (2)	0.05848 (14)	0.0361 (7)	
C36	0.1413 (3)	0.1945 (2)	0.02003 (14)	0.0396 (7)	
H36	0.1330	0.1769	−0.0172	0.047*	
C37	0.0960 (3)	0.2754 (2)	0.03776 (15)	0.0396 (7)	
C38	0.1059 (3)	0.3019 (2)	0.09184 (15)	0.0377 (7)	
H38	0.0721	0.3570	0.1033	0.045*	
N1	0.3352 (2)	0.32700 (16)	0.34911 (10)	0.0269 (5)	
N2	0.6672 (2)	0.38628 (17)	0.33856 (10)	0.0271 (5)	
H1N	0.651 (4)	0.440 (3)	0.3363 (14)	0.033*	
N3	0.4523 (2)	0.13695 (16)	0.27536 (10)	0.0278 (5)	
H2N	0.510 (4)	0.166 (2)	0.2945 (15)	0.033*	
O2	0.58152 (19)	0.24951 (13)	0.34972 (9)	0.0289 (4)	
O3	0.4177 (2)	0.06170 (14)	0.19576 (9)	0.0328 (5)	
O4	0.2526 (2)	0.22167 (14)	0.21627 (9)	0.0322 (4)	
O5	0.1021 (2)	0.32785 (16)	0.20878 (10)	0.0423 (6)	
Cl1	0.96231 (8)	0.07105 (6)	0.29804 (4)	0.0481 (2)	
Cl2	0.85139 (8)	0.14928 (6)	0.08777 (4)	0.0480 (2)	
Cl3	0.24876 (9)	0.03602 (6)	0.03774 (4)	0.0465 (2)	
Cl4	0.01738 (10)	0.34193 (6)	−0.00931 (4)	0.0512 (2)	

### (II) (3S,4aS,8aS)-2-[(2R,3S)-3-(2,5-Dichlorobenzamido)-2-(2,5-dichlorobenzoyloxy)-4-phenylbutyl]-N-tert-butyldecahydroisoquinoline-3-carboxamide Atomic displacement parameters (Å^2^)

**Table:**

	*U*^11^	*U*^22^	*U*^33^	*U*^12^	*U*^13^	*U*^23^
C1	0.0196 (12)	0.0323 (15)	0.0355 (15)	0.0010 (11)	−0.0018 (12)	−0.0014 (12)
C2	0.0208 (13)	0.0339 (15)	0.0352 (15)	−0.0008 (12)	−0.0024 (12)	−0.0035 (12)
C3	0.0211 (13)	0.0293 (14)	0.0370 (15)	−0.0019 (11)	0.0016 (12)	−0.0011 (12)
C4	0.0298 (15)	0.0296 (15)	0.0395 (16)	−0.0019 (12)	0.0019 (13)	−0.0014 (13)
C5	0.0230 (14)	0.0251 (13)	0.0369 (15)	0.0010 (11)	−0.0014 (12)	−0.0036 (12)
C6	0.0234 (14)	0.0331 (16)	0.0452 (17)	0.0005 (13)	−0.0064 (13)	0.0006 (13)
C7	0.0178 (13)	0.0267 (14)	0.0362 (15)	−0.0012 (11)	0.0003 (11)	−0.0007 (11)
C8	0.0228 (13)	0.0319 (15)	0.0364 (15)	−0.0038 (12)	−0.0005 (12)	−0.0019 (12)
C9	0.0299 (15)	0.0318 (15)	0.0365 (16)	−0.0012 (13)	0.0037 (13)	0.0006 (12)
C10	0.0424 (18)	0.0403 (18)	0.0370 (16)	−0.0024 (14)	0.0042 (14)	−0.0017 (14)
C11	0.054 (2)	0.047 (2)	0.0358 (17)	−0.0020 (16)	0.0048 (15)	0.0032 (15)
C12	0.052 (2)	0.052 (2)	0.048 (2)	−0.0100 (18)	0.0109 (17)	0.0066 (17)
C13	0.0362 (17)	0.0417 (18)	0.0442 (18)	−0.0095 (14)	0.0065 (15)	0.0009 (15)
C14	0.0253 (14)	0.0387 (17)	0.0401 (17)	0.0016 (13)	0.0059 (12)	−0.0006 (13)
C15	0.0199 (14)	0.0337 (16)	0.0417 (17)	0.0023 (12)	0.0016 (12)	−0.0036 (13)
C16	0.0180 (12)	0.0292 (15)	0.0292 (13)	−0.0016 (11)	−0.0014 (10)	0.0004 (11)
C17	0.0180 (13)	0.0272 (14)	0.0480 (17)	−0.0006 (11)	0.0045 (12)	0.0042 (13)
C18	0.0219 (15)	0.046 (2)	0.088 (3)	−0.0029 (14)	−0.0063 (17)	0.026 (2)
C19	0.049 (2)	0.044 (2)	0.051 (2)	−0.0072 (16)	0.0195 (17)	−0.0077 (16)
C20	0.0225 (14)	0.0325 (16)	0.0557 (19)	−0.0026 (12)	0.0025 (14)	0.0076 (14)
C21	0.0472 (19)	0.0304 (16)	0.0358 (16)	−0.0009 (15)	0.0006 (14)	0.0002 (13)
C22	0.049 (2)	0.0421 (19)	0.0438 (19)	0.0047 (16)	−0.0052 (16)	0.0011 (15)
C23	0.062 (3)	0.053 (2)	0.052 (2)	0.012 (2)	−0.006 (2)	0.0051 (18)
C24	0.096 (4)	0.042 (2)	0.048 (2)	0.016 (2)	−0.006 (2)	0.0015 (17)
C25	0.091 (4)	0.0340 (19)	0.056 (2)	−0.012 (2)	−0.006 (2)	−0.0001 (17)
C26	0.066 (3)	0.0379 (19)	0.0471 (19)	−0.0139 (18)	−0.0119 (18)	0.0062 (16)
C27	0.0217 (14)	0.0256 (14)	0.0416 (16)	0.0008 (11)	0.0001 (12)	−0.0066 (12)
C28	0.0255 (15)	0.0314 (15)	0.0437 (17)	0.0010 (12)	0.0003 (12)	−0.0056 (13)
C29	0.0214 (14)	0.0329 (16)	0.0546 (19)	0.0036 (12)	−0.0048 (14)	−0.0096 (14)
C30	0.0255 (15)	0.0283 (15)	0.062 (2)	0.0003 (12)	0.0058 (14)	−0.0119 (14)
C31	0.0311 (16)	0.0308 (16)	0.0476 (18)	−0.0032 (12)	0.0060 (14)	−0.0076 (14)
C32	0.0256 (14)	0.0263 (14)	0.0436 (17)	−0.0019 (12)	0.0003 (13)	−0.0057 (12)
C33	0.0241 (14)	0.0340 (16)	0.0411 (16)	−0.0003 (13)	−0.0041 (13)	0.0017 (13)
C34	0.0244 (14)	0.0362 (16)	0.0418 (16)	0.0002 (12)	−0.0024 (12)	0.0021 (14)
C35	0.0332 (16)	0.0354 (16)	0.0397 (16)	−0.0014 (13)	−0.0010 (13)	0.0010 (13)
C36	0.0371 (18)	0.0451 (18)	0.0365 (17)	−0.0038 (15)	−0.0013 (14)	0.0021 (14)
C37	0.0325 (16)	0.0402 (18)	0.0460 (18)	−0.0040 (14)	−0.0052 (14)	0.0090 (15)
C38	0.0287 (15)	0.0354 (17)	0.0491 (19)	−0.0012 (13)	−0.0061 (14)	0.0028 (14)
N1	0.0177 (11)	0.0294 (12)	0.0336 (12)	−0.0025 (10)	0.0009 (10)	−0.0021 (10)
N2	0.0201 (11)	0.0249 (12)	0.0364 (13)	−0.0005 (10)	0.0006 (10)	0.0002 (10)
N3	0.0198 (11)	0.0277 (12)	0.0358 (13)	0.0006 (10)	−0.0018 (10)	−0.0039 (10)
O2	0.0217 (9)	0.0253 (10)	0.0395 (11)	−0.0011 (8)	−0.0028 (8)	0.0001 (8)
O3	0.0254 (10)	0.0300 (11)	0.0432 (12)	0.0010 (8)	−0.0046 (9)	−0.0076 (9)
O4	0.0250 (10)	0.0356 (11)	0.0361 (11)	0.0038 (9)	−0.0054 (9)	−0.0030 (9)
O5	0.0332 (11)	0.0429 (13)	0.0507 (14)	0.0121 (10)	−0.0066 (10)	−0.0081 (11)
Cl1	0.0266 (4)	0.0544 (5)	0.0632 (5)	0.0083 (4)	−0.0099 (4)	−0.0119 (4)
Cl2	0.0419 (4)	0.0490 (5)	0.0529 (5)	−0.0093 (4)	0.0139 (4)	−0.0051 (4)
Cl3	0.0514 (5)	0.0428 (4)	0.0452 (4)	0.0061 (4)	0.0023 (4)	−0.0044 (3)
Cl4	0.0557 (5)	0.0457 (5)	0.0523 (5)	0.0007 (4)	−0.0155 (4)	0.0113 (4)

### (II) (3S,4aS,8aS)-2-[(2R,3S)-3-(2,5-Dichlorobenzamido)-2-(2,5-dichlorobenzoyloxy)-4-phenylbutyl]-N-tert-butyldecahydroisoquinoline-3-carboxamide Geometric parameters (Å, º)

**Table:**

C1—N1	1.463 (4)	C17—N2	1.473 (4)
C1—C2	1.522 (4)	C17—C18	1.512 (5)
C1—H1A	0.9900	C17—C20	1.527 (4)
C1—H1B	0.9900	C17—C19	1.530 (5)
C2—O4	1.453 (4)	C18—H18A	0.9800
C2—C3	1.540 (4)	C18—H18B	0.9800
C2—H2	1.0000	C18—H18C	0.9800
C3—N3	1.462 (4)	C19—H19A	0.9800
C3—C4	1.537 (4)	C19—H19B	0.9800
C3—H3	1.0000	C19—H19C	0.9800
C4—C21	1.511 (4)	C20—H20A	0.9800
C4—H4A	0.9900	C20—H20B	0.9800
C4—H4B	0.9900	C20—H20C	0.9800
C5—O3	1.238 (4)	C21—C26	1.376 (5)
C5—N3	1.335 (4)	C21—C22	1.405 (5)
C5—C27	1.505 (4)	C22—C23	1.391 (5)
C6—O5	1.206 (4)	C22—H22	0.9500
C6—O4	1.348 (4)	C23—C24	1.364 (7)
C6—C33	1.491 (5)	C23—H23	0.9500
C7—N1	1.467 (3)	C24—C25	1.374 (8)
C7—C16	1.526 (4)	C24—H24	0.9500
C7—C8	1.532 (4)	C25—C26	1.416 (6)
C7—H7	1.0000	C25—H25	0.9500
C8—C9	1.530 (4)	C26—H26	0.9500
C8—H8A	0.9900	C27—C32	1.389 (5)
C8—H8B	0.9900	C27—C28	1.395 (4)
C9—C10	1.527 (4)	C28—C29	1.395 (4)
C9—C14	1.540 (4)	C28—H28	0.9500
C9—H9	1.0000	C29—C30	1.379 (5)
C10—C11	1.523 (5)	C29—Cl1	1.735 (3)
C10—H10A	0.9900	C30—C31	1.393 (5)
C10—H10B	0.9900	C30—H30	0.9500
C11—C12	1.525 (6)	C31—C32	1.391 (4)
C11—H11A	0.9900	C31—Cl2	1.731 (4)
C11—H11B	0.9900	C32—H32	0.9500
C12—C13	1.534 (5)	C33—C38	1.394 (5)
C12—H12A	0.9900	C33—C34	1.397 (5)
C12—H12B	0.9900	C34—C35	1.383 (5)
C13—C14	1.522 (5)	C34—H34	0.9500
C13—H13A	0.9900	C35—C36	1.388 (5)
C13—H13B	0.9900	C35—Cl3	1.740 (3)
C14—C15	1.521 (4)	C36—C37	1.385 (5)
C14—H14	1.0000	C36—H36	0.9500
C15—N1	1.470 (4)	C37—C38	1.382 (5)
C15—H15A	0.9900	C37—Cl4	1.736 (3)
C15—H15B	0.9900	C38—H38	0.9500
C16—O2	1.247 (4)	N2—H1N	0.84 (4)
C16—N2	1.335 (4)	N3—H2N	0.88 (4)
			
N1—C1—C2	111.1 (2)	N2—C17—C20	106.8 (2)
N1—C1—H1A	109.4	C18—C17—C20	109.6 (3)
C2—C1—H1A	109.4	N2—C17—C19	108.5 (3)
N1—C1—H1B	109.4	C18—C17—C19	111.4 (3)
C2—C1—H1B	109.4	C20—C17—C19	109.4 (3)
H1A—C1—H1B	108.0	C17—C18—H18A	109.5
O4—C2—C1	108.1 (2)	C17—C18—H18B	109.5
O4—C2—C3	103.1 (2)	H18A—C18—H18B	109.5
C1—C2—C3	116.5 (2)	C17—C18—H18C	109.5
O4—C2—H2	109.6	H18A—C18—H18C	109.5
C1—C2—H2	109.6	H18B—C18—H18C	109.5
C3—C2—H2	109.6	C17—C19—H19A	109.5
N3—C3—C4	109.5 (2)	C17—C19—H19B	109.5
N3—C3—C2	110.0 (2)	H19A—C19—H19B	109.5
C4—C3—C2	114.9 (2)	C17—C19—H19C	109.5
N3—C3—H3	107.4	H19A—C19—H19C	109.5
C4—C3—H3	107.4	H19B—C19—H19C	109.5
C2—C3—H3	107.4	C17—C20—H20A	109.5
C21—C4—C3	111.2 (3)	C17—C20—H20B	109.5
C21—C4—H4A	109.4	H20A—C20—H20B	109.5
C3—C4—H4A	109.4	C17—C20—H20C	109.5
C21—C4—H4B	109.4	H20A—C20—H20C	109.5
C3—C4—H4B	109.4	H20B—C20—H20C	109.5
H4A—C4—H4B	108.0	C26—C21—C22	118.2 (3)
O3—C5—N3	124.7 (3)	C26—C21—C4	122.0 (3)
O3—C5—C27	120.0 (3)	C22—C21—C4	119.7 (3)
N3—C5—C27	115.3 (3)	C23—C22—C21	121.2 (4)
O5—C6—O4	124.5 (3)	C23—C22—H22	119.4
O5—C6—C33	124.9 (3)	C21—C22—H22	119.4
O4—C6—C33	110.5 (3)	C24—C23—C22	119.9 (4)
N1—C7—C16	111.3 (2)	C24—C23—H23	120.0
N1—C7—C8	109.8 (2)	C22—C23—H23	120.0
C16—C7—C8	109.1 (2)	C23—C24—C25	120.4 (4)
N1—C7—H7	108.9	C23—C24—H24	119.8
C16—C7—H7	108.9	C25—C24—H24	119.8
C8—C7—H7	108.9	C24—C25—C26	120.1 (4)
C9—C8—C7	111.1 (2)	C24—C25—H25	120.0
C9—C8—H8A	109.4	C26—C25—H25	120.0
C7—C8—H8A	109.4	C21—C26—C25	120.3 (4)
C9—C8—H8B	109.4	C21—C26—H26	119.9
C7—C8—H8B	109.4	C25—C26—H26	119.9
H8A—C8—H8B	108.0	C32—C27—C28	121.0 (3)
C10—C9—C8	113.7 (3)	C32—C27—C5	117.6 (3)
C10—C9—C14	111.0 (3)	C28—C27—C5	121.4 (3)
C8—C9—C14	110.0 (2)	C29—C28—C27	118.5 (3)
C10—C9—H9	107.3	C29—C28—H28	120.7
C8—C9—H9	107.3	C27—C28—H28	120.7
C14—C9—H9	107.3	C30—C29—C28	121.7 (3)
C11—C10—C9	112.7 (3)	C30—C29—Cl1	119.2 (2)
C11—C10—H10A	109.1	C28—C29—Cl1	119.1 (3)
C9—C10—H10A	109.1	C29—C30—C31	118.6 (3)
C11—C10—H10B	109.1	C29—C30—H30	120.7
C9—C10—H10B	109.1	C31—C30—H30	120.7
H10A—C10—H10B	107.8	C32—C31—C30	121.3 (3)
C10—C11—C12	111.5 (3)	C32—C31—Cl2	119.2 (3)
C10—C11—H11A	109.3	C30—C31—Cl2	119.4 (3)
C12—C11—H11A	109.3	C27—C32—C31	118.9 (3)
C10—C11—H11B	109.3	C27—C32—H32	120.6
C12—C11—H11B	109.3	C31—C32—H32	120.6
H11A—C11—H11B	108.0	C38—C33—C34	120.8 (3)
C11—C12—C13	110.8 (3)	C38—C33—C6	118.8 (3)
C11—C12—H12A	109.5	C34—C33—C6	120.3 (3)
C13—C12—H12A	109.5	C35—C34—C33	118.3 (3)
C11—C12—H12B	109.5	C35—C34—H34	120.8
C13—C12—H12B	109.5	C33—C34—H34	120.8
H12A—C12—H12B	108.1	C34—C35—C36	122.3 (3)
C14—C13—C12	110.6 (3)	C34—C35—Cl3	119.3 (3)
C14—C13—H13A	109.5	C36—C35—Cl3	118.3 (3)
C12—C13—H13A	109.5	C37—C36—C35	117.8 (3)
C14—C13—H13B	109.5	C37—C36—H36	121.1
C12—C13—H13B	109.5	C35—C36—H36	121.1
H13A—C13—H13B	108.1	C38—C37—C36	122.1 (3)
C15—C14—C13	112.6 (3)	C38—C37—Cl4	119.7 (3)
C15—C14—C9	109.5 (2)	C36—C37—Cl4	118.2 (3)
C13—C14—C9	112.0 (3)	C37—C38—C33	118.7 (3)
C15—C14—H14	107.5	C37—C38—H38	120.6
C13—C14—H14	107.5	C33—C38—H38	120.6
C9—C14—H14	107.5	C1—N1—C7	112.5 (2)
N1—C15—C14	112.9 (3)	C1—N1—C15	108.8 (2)
N1—C15—H15A	109.0	C7—N1—C15	110.1 (2)
C14—C15—H15A	109.0	C16—N2—C17	126.5 (3)
N1—C15—H15B	109.0	C16—N2—H1N	118 (3)
C14—C15—H15B	109.0	C17—N2—H1N	114 (3)
H15A—C15—H15B	107.8	C5—N3—C3	123.4 (3)
O2—C16—N2	123.9 (3)	C5—N3—H2N	118 (2)
O2—C16—C7	122.1 (2)	C3—N3—H2N	119 (2)
N2—C16—C7	114.0 (2)	C6—O4—C2	119.3 (2)
N2—C17—C18	111.1 (3)		
			
N1—C1—C2—O4	175.5 (2)	C27—C28—C29—Cl1	179.7 (2)
N1—C1—C2—C3	−69.1 (3)	C28—C29—C30—C31	−1.6 (5)
O4—C2—C3—N3	68.7 (3)	Cl1—C29—C30—C31	179.8 (2)
C1—C2—C3—N3	−49.5 (3)	C29—C30—C31—C32	0.5 (5)
O4—C2—C3—C4	−167.3 (2)	C29—C30—C31—Cl2	−178.7 (2)
C1—C2—C3—C4	74.4 (3)	C28—C27—C32—C31	−1.6 (5)
N3—C3—C4—C21	−59.4 (3)	C5—C27—C32—C31	178.7 (3)
C2—C3—C4—C21	176.4 (3)	C30—C31—C32—C27	1.1 (5)
N1—C7—C8—C9	−58.6 (3)	Cl2—C31—C32—C27	−179.7 (2)
C16—C7—C8—C9	179.2 (2)	O5—C6—C33—C38	15.3 (5)
C7—C8—C9—C10	179.9 (3)	O4—C6—C33—C38	−167.0 (3)
C7—C8—C9—C14	54.7 (3)	O5—C6—C33—C34	−159.9 (3)
C8—C9—C10—C11	−72.6 (4)	O4—C6—C33—C34	17.8 (4)
C14—C9—C10—C11	52.1 (4)	C38—C33—C34—C35	−1.6 (5)
C9—C10—C11—C12	−53.9 (4)	C6—C33—C34—C35	173.5 (3)
C10—C11—C12—C13	55.9 (4)	C33—C34—C35—C36	2.6 (5)
C11—C12—C13—C14	−57.1 (4)	C33—C34—C35—Cl3	−175.4 (2)
C12—C13—C14—C15	−179.9 (3)	C34—C35—C36—C37	−1.5 (5)
C12—C13—C14—C9	56.2 (4)	Cl3—C35—C36—C37	176.5 (3)
C10—C9—C14—C15	−179.2 (3)	C35—C36—C37—C38	−0.7 (5)
C8—C9—C14—C15	−52.4 (3)	C35—C36—C37—Cl4	−177.4 (3)
C10—C9—C14—C13	−53.5 (4)	C36—C37—C38—C33	1.7 (5)
C8—C9—C14—C13	73.3 (3)	Cl4—C37—C38—C33	178.3 (2)
C13—C14—C15—N1	−69.1 (3)	C34—C33—C38—C37	−0.5 (5)
C9—C14—C15—N1	56.2 (3)	C6—C33—C38—C37	−175.6 (3)
N1—C7—C16—O2	−35.0 (4)	C2—C1—N1—C7	153.7 (2)
C8—C7—C16—O2	86.4 (3)	C2—C1—N1—C15	−84.0 (3)
N1—C7—C16—N2	147.1 (2)	C16—C7—N1—C1	−57.2 (3)
C8—C7—C16—N2	−91.6 (3)	C8—C7—N1—C1	−178.2 (2)
C3—C4—C21—C26	−85.2 (4)	C16—C7—N1—C15	−178.8 (2)
C3—C4—C21—C22	90.5 (4)	C8—C7—N1—C15	60.3 (3)
C26—C21—C22—C23	1.5 (5)	C14—C15—N1—C1	175.6 (2)
C4—C21—C22—C23	−174.3 (3)	C14—C15—N1—C7	−60.7 (3)
C21—C22—C23—C24	0.4 (6)	O2—C16—N2—C17	4.7 (5)
C22—C23—C24—C25	−2.0 (6)	C7—C16—N2—C17	−177.5 (3)
C23—C24—C25—C26	1.8 (7)	C18—C17—N2—C16	−53.9 (4)
C22—C21—C26—C25	−1.7 (5)	C20—C17—N2—C16	−173.3 (3)
C4—C21—C26—C25	174.0 (4)	C19—C17—N2—C16	68.9 (4)
C24—C25—C26—C21	0.1 (6)	O3—C5—N3—C3	0.8 (5)
O3—C5—C27—C32	42.1 (4)	C27—C5—N3—C3	180.0 (3)
N3—C5—C27—C32	−137.1 (3)	C4—C3—N3—C5	136.6 (3)
O3—C5—C27—C28	−137.6 (3)	C2—C3—N3—C5	−96.3 (3)
N3—C5—C27—C28	43.2 (4)	O5—C6—O4—C2	4.8 (5)
C32—C27—C28—C29	0.5 (5)	C33—C6—O4—C2	−172.9 (2)
C5—C27—C28—C29	−179.8 (3)	C1—C2—O4—C6	−78.2 (3)
C27—C28—C29—C30	1.2 (5)	C3—C2—O4—C6	158.0 (2)

### (II) (3S,4aS,8aS)-2-[(2R,3S)-3-(2,5-Dichlorobenzamido)-2-(2,5-dichlorobenzoyloxy)-4-phenylbutyl]-N-tert-butyldecahydroisoquinoline-3-carboxamide Hydrogen-bond geometry (Å, º)

**Table:**

*D*—H···*A*	*D*—H	H···*A*	*D*···*A*	*D*—H···*A*
N2—H1*N*···O3^i^	0.84 (4)	2.13 (4)	2.931 (3)	160 (3)
N3—H2*N*···O2	0.88 (4)	1.99 (4)	2.834 (3)	159 (3)
C4—H4*A*···N1	0.99	2.55	3.149 (4)	119
C18—H18*A*···O2	0.98	2.36	2.975 (4)	120
C34—H34···O3	0.95	2.40	3.324 (4)	163

Symmetry code: (i) −*x*+1, *y*+1/2, −*z*+1/2.
